# Visceral Leishmaniasis, Northern Somalia, 2013–2019

**DOI:** 10.3201/eid2601.181851

**Published:** 2020-01

**Authors:** Mikko K. Aalto, Temmy Sunyoto, Mohamed Ahmed Ali Yusuf, Abdiaziz Ahmed Mohamed, Gert Van der Auwera, Jean-Claude Dujardin

**Affiliations:** Bosaso General Hospital, Bosaso, Somalia (M.K. Aalto, M.A.A. Yusuf, A.A. Mohamed);; Institute of Tropical Medicine, Antwerp, Belgium (T. Sunyoto, G. Van der Auwera, J.-C. Dujardin)

**Keywords:** Leishmania, visceral leishmaniasis, Somalia, promastigote, parasites, Leishmania donovani

## Abstract

We identified visceral leishmaniasis caused by *Leishmania donovani* in a previously unknown focus in northern Somalia. Clinical and epidemiologic characteristics of 118 cases during 2013–2019 in Bosaso, the region’s commercial capital, have raised suspicion of visceral leishmaniasis endemicity status there.

Visceral leishmaniasis (VL), the fatal form of a parasitic disease caused by *Leishmania donovani* complex, has been known to exist in southern Somalia since the 1930s, but its presence in the northern part of the country is unclear (*1*–*4*). We report VL existence through initial investigation of suspected case-patients in Bosaso General Hospital (Bosaso, Somalia) during December 2013–February 2019. Bosaso is a city in the northeastern Bari Province of Somalia, which serves as the region’s commercial capital and is a major seaport on the southern coast of the Gulf of Aden.

Clinicians suspected VL disease in 2013 in several infants with extreme wasting, splenomegaly, pancytopenia, and death. In previous years, leukemia was misdiagnosed in such children. VL was eventually confirmed through microscopic demonstration of *Leishmania* amastigotes in bone marrow and spleen aspirates. Furthermore, these patients responded well when empirically treated with sodium stibogluconate, the mainstay of VL therapy in eastern Africa.

Since then, the hospital’s clinicians have maintained a database of patients with suspected and confirmed VL (case definition: fever >2 weeks’ duration, splenomegaly, wasting, and pancytopenia). A total of 118 patients were confirmed to have VL during 2013–2019 by microscopy, in vitro culture (introduced in 2016 [Appendix]), or serology. After Bosaso General Hospital reported the first cases to the World Health Organization (WHO) in 2014, WHO provided rK39 rapid diagnostic tests (Kalazar Detect; Inbios, https://inbios.com/kalazar-detecttm-rapid-test-for-visceral-leishmaniasis-intl) for use in accordance with Somalia’s national leishmaniasis guidelines of 2012 (*5*).

Among the 118 identified patients, nearly all (107 [91%]) were children. Patients’ ages ranged from 6 months to 60 years; 78 (66%) were male. The most frequent symptoms were wasting, splenomegaly, and severe or moderate pancytopenia, along with persistent fever. Lymphadenopathy was absent, as was post–kala-azar dermal leishmaniasis, although this condition might have been missed because no follow-up system was in place. Whenever possible, patients with confirmed VL were treated with sodium stibogluconate (20 mg/kg) combined with paromomycin (20 mg/kg), both intramuscularly. However, because of the low availability of paromomycin, sodium stibogluconate monotherapy was administered when paromomycin was unavailable. Outcome records were available for 103 (87%) patients, 85 (85%) of whom were clinically cured. Eighteen (17%) patients died. As part of routine data collection, the patients’ origins were documented: all patients came from Sanaag and Bari regions in northern Somalia and had no history of traveling outside this area.

For 3 patients, we attempted to identify *Leishmania* species by extracting DNA from microscopy slides and sequencing partial fragments of the PCR-amplified HSP70 gene (*6*). In 2 patients, *L. donovani* was identified (European Nucleotide Archive study PRJEB34786, accession nos. LR723650 and LR723651 [Appendix, section 1]).

Suspected VL patients whose illnesses fit the case definition first underwent rapid diagnostic testing to exclude malaria (*5*). During 2013–2019, only 2 were found positive for malaria, which was confirmed by microscopy. Both also had confirmed VL and subsequently received antimalarial and antileishmanial therapy. In accordance with national guidelines (*5*), patients also were tested for HIV; no VL patient was positive. Screening for other underlying conditions was undertaken when feasible; for example, with chest radiograph. One VL case-patient with concomitant pulmonary tuberculosis was referred to the tuberculosis center after finishing antileishmanial treatment.

We describe a previously unreported focus of VL in northern Somalia. The age distribution of the case-patients indicates that VL seems to be endemic in this region; it is unlikely that all cases were imported or present as an “outbreak” such as that described in Huddur (Bakool region) in 2001 (*7*). From the perspectives of families of the patients and the local health workers, the disease has been known in this area for years. Despite ongoing war and unrest in southern Somalia and the prevalence of displacement in the country, it appears implausible that the VL patients in Bosaso came from there. The nearest known focus is across the Gulf of Aden, in southern Yemen (*8*,*9*), where Somali diaspora is present. Because sea travel with small wooden boats is common across the gulf and has been for centuries, this focus might play a role in the cases described here and merits further exploration. Surveillance for VL should be strengthened in northern Somalia, and access to adequate diagnosis and treatment must be provided to reduce transmission, illness, and death. Support and collaboration across stakeholders, including WHO and national health actors, must be continued to tackle the disease in a comprehensive manner. Further investigation (e.g., a cross-sectional survey) might be considered to define the infection rate in this newly identified focus and determine its level of endemicity.

Appendix Additional methods for determining visceral leishmaniasis, northern Somalia, 2013–2019.
