# Supplementary material for: Visceral Leishmaniasis, Northern Somalia, 2013–2019
Source: Emerg Infect Dis. 2020 Jan;26(1):153–4. doi: 10.3201/eid2601.181851 (PMC6924893; doi:10.3201/eid2601.181851)
Supplement: Appendix — Additional methods for determining visceral leishmaniasis, northern Somalia, 2013–2019. [file 18-1851-Techapp-s1.pdf]

Article DOI: https://doi.org/10.3201/eid2601.181851

Visceral Leishmaniasis, Northern Somalia, 2013–2019

Appendix

1. Short Summary on Methods of HSP70 Identification and Molecular Typing

We identified *L. donovani* in 2 samples coming from different patients

BGH 201812 03

BGH 201812 23

As described elsewhere (*1*), two partial *Leishmania* HSP70 coding sequences were amplified in both samples with the following HSP70 primers:

Fragment N: HSP70-F25 5′ GGACGCCGGCACGATTKCT 3′

HSP70-R617 5′ CGAAGAAGTCCGATACGAGGGA 3′

Fragment T: HSP70–6F 5′ GTGCACGACGTGGTGCTGGTG 3′

HSP70-R1310 5′ CCTGGTTGTTGTTCAGCCACTC 3′

The PCR fragments were sequenced from both sides. As the fragments overlap, one sequence contig was constructed for each of both samples, and the sequences were found identical.

Sequences were deposited at European Nucleotide Archive (ENA)

Study accession number in the European Nucleotide Archive is PRJEB34786

https://www.ebi.ac.uk/ena/data/view/PRJEB34786

Accession numbers of both sequences are:

LR723650: MHOM/SO/2018/BGH201812_03

LR723651: MHOM/SO/2018/BGH201812_23

https://www.ebi.ac.uk/ena/data/view/LR723650-LR723651

The sequences were used to construct the dendrogram (Appendix Figure), as described elsewhere (*1*). The two Somali samples (red square) cluster clearly with *L. donovani* strains.

2. In Vitro Culture Methods.

Leishmania culture was performed in vials of tryptone yeast extract agar with 5% human blood at 25°C until positive or for 30 days.

Reference

1. Van der Auwera G, Maes I, De Doncker S, Ravel C, Cnops L, Van Esbroeck M, et al. Heat-shock protein 70 gene sequencing for *Leishmania* species typing in European tropical infectious disease clinics. Euro Surveill. 2013;18:20543. PubMed http://dx.doi.org/10.2807/1560-7917.ES2013.18.30.20543
